# Soy-Based Adhesive Cross-Linked by Phenol-Formaldehyde-Glutaraldehyde

**DOI:** 10.3390/polym9050169

**Published:** 2017-05-08

**Authors:** Zhigang Wu, Xuedong Xi, Hong Lei, Guanben Du

**Affiliations:** 1 Yunnan Provincial Key Laboratory of Wood Adhesives and Glued Products, Southwest Forestry University, Kunming 650224, Yunnan, China; wzhigang9@163.com (Z.W.); xuedongjx@163.com (X.X.); 2 College of Forestry, Guizhou University, Guiyang 550025, Guizhou, China

**Keywords:** soy-based adhesive, glutaraldehyde, phenol-formaldehyde, preparation procedure, crosslinking modification

## Abstract

To prepare a low-formaldehyde soy-based adhesive with good water resistance, phenol-formaldehyde modified with glutaraldehyde (PFG) with lower free phenol and free formaldehyde contents was used to cross-link the soy-based adhesive. The results showed that the mechanical properties and water resistance of plywood prepared with soy-based adhesive with PFG was better than that of plywood with the same amount of phenol-formaldehyde (PF). The reaction between phenol and glutaraldehyde was proved by ^13^C-NMR. Under the optimized preparation conditions for plywood, that is to say, press temperature 160 °C, press time 4 min and resin loading 320 g·m^−2^, type I plywood could be prepared with 9% PFG as a cross-linker of soy-based adhesive. The Differential Scanning Calorimetry (DSC) result confirmed the cross-linking reaction between soy-based adhesive and PFG or PF. The activation energy of soy-based adhesive with cross-linker PFG was higher than that with PF resin.

## 1. Introduction 

With the mounting needs for environment-friendly adhesives in the wood industry, the development of bio-based adhesives has become an important practice. The main bio-materials used in the preparation of bio-based adhesives include starch, soy flour, tannin, lignin, and so on. Although there is a long history of using soy flour with high protein content to prepare soy-based adhesive, soy-based adhesive had once been pushed out of the wood industry for its poor water resistance. Actually, today most of the studies on this adhesive focus on the improvement of its water resistance [1,2,3,4]. Cross-linking modification has been proved to be one of the effective modification methods to resolve this problem. Some cross-linkers used for this modification include melamine-urea-formaldehyde resin [5,6,7], epichlorohydrin [8], 1,3-dichloro-2-propanol [9], aldehyde and its derivatives [10], and so on. Phenol-formaldehyde (PF) resin, a widely-used exterior wood adhesive, can also be used as a cross-linker for soy-based adhesive [11]. Kreibich used soy adhesive and a phenol-resorcinol-formaldehyde (PRF) adhesive to prepare finger-jointed boards with a “honeymoon” process [12]. Yang developed a soy-based adhesive with PF for plywood [13]. In this formulation, the solid weight ratio of PF to soy was 30:70. Another similar soy-based adhesive for Oriented Strand Board (OSB) was introduced by Hse [14]. Because of the toxicity of phenol and formaldehyde, it is obvious that the addition of PF has to be as little as possible, although cured PF resin without free phenol and formaldehyde shows good water and weather resistance and can be used safely.

Glutaraldehyde is a linear 5-carbon dialdehyde. Compared with formaldehyde, glutaraldehyde shows low or no danger to the human body. The toxicological effects on animals exposed to glutaraldehyde are mainly irritation at the site of contact, and contact sensitization [15]. At the same time, since glutaraldehyde with high reactivity can react with several functional groups of proteins, such as amine, thiol, phenol, and imidazole, it is widely used as a protein cross-linker. In fact, some study has showed that it is the most effective cross-linker in monoaldehyde (formadehyde) and dialdehydes having chain lengths of two to six carbon atoms (glyoxal, malonaldehyde, succinaldehyde, glutaraldehyde, and adipaldehyde) when used to cross-link collagen [16]. In this work, glutaraldehyde was firstly reacted with phenol formaldehyde. The resulted phenol-formaldehyde-glutaraldehyde (PFG) was then used as a cross-linker for the soy protein-based adhesive. The objective of this work was as follows: (1) To decease the addition amount of the cross-linker; and (2) to decrease the formaldehyde content in the soy-based adhesive due to the added cross-linker.

## 2. Materials and Methods

### 2.1. Materials

Defatted soy flour (53.4% protein content) was obtained from Yuxin Soybean Protein Co., Ltd., Qingdao, Shandong, China. The thickness of the Poplar veneer was 1.5 mm and its moisture content was 8–10%. The formaldehyde 37 wt % and glutaraldehyde 50 wt % were obtained from Sinopharm Chemical Reagent Co., Ltd., Beijing, China. All other chemicals in this work were reagent grade.

### 2.2. Preparation of Phenolic Resin-Based Cross-Linkers and Test of Their Performances

The PF resin control with molar ratio F:P = 1.8 was prepared as follows: 94 parts by mass phenol and 146 parts formaldehyde 37% were put into a 500-mL flat-bottom flask, which was equipped with a condenser, thermometer and mechanical stirrer. NaOH 30% was used to adjust the pH to 9–9.5. After stirring well for several minutes, the temperature of the mixture was slowly increased to 85–90 °C. After 60 min at this temperature, PF resin control was obtained and then cooled to room temperature.

Glutaraldehyde 50% was used as a modifier of phenol-formaldehyde resin to prepare PFG with a final molar ratio F:G:P = 1.5:0.3:1. At the beginning of the preparation, the total amount of glutaraldehyde was charged to a flask together with phenol and formaldehyde 37%. The preparation procedure of PFG was the same as that of the PF control.

The phenol-glutaraldehyde (PG) resin with molar ratio G:P = 1.0 was prepared as follows: 94 parts by mass phenol was put into a 500-mL flat-bottom flask, which was equipped with a condenser, thermometer and mechanical stirrer. NaOH 30% was used to adjust the pH to 9–9.5. The temperature was slowly increased to 92–94 °C. 188 parts of glutaraldehyde 50% was continuously charged to the flask. After 3.5 h at this temperature, PG resin was achieved and then cooled to room temperature.

The content of free formaldehyde and free phenol of PF and PFG was tested with the method introduced by the Chinese national standard GB/T 9846.3-2004.

### 2.3. Preparation of Soy-Based Adhesive

Soy-based adhesive was prepared according to a method already reported [11]: 320 parts by mass water and 80 parts soy flour were added to a three-neck round-bottom flask, which was equipped with a mechanical stirrer, thermometer and condenser. With continuously stirring, the mixture was heated to 45 °C. Then 21.3 parts NaOH 30% was added to the flask. After 30 min, 20 parts 40% urea solution was added. After another 20 min, the soy-based adhesive with the name of S was achieved. Its solid content was 24% ± 1%.

### 2.4. Preparation of Plywood Samples Bonded with Soy-Based Adhesive

The soy-based adhesive was mixed mechanically with PF and PFG and then was immediately brushed on the surface of the veneers to prepare the three-layer plywood of dimensions 300 mm × 220 mm × 4 mm. The addition amount of PF or PFG was 9% to 15% of the solid soy-based adhesive. With the double sided resin loading 320 g·m^−2^, the plywood mat was prepared. The veneers with adhesives rested at room temperature for 15–20 min and then were assembled to be sent into the press. The plywood was prepared under press pressure 1.5 MPa at 180 °C for 3 min.

To optimize the preparation procedure of plywood with soy-based adhesive and to reduce the number of experiments, orthogonal experiments were conducted. The orthogonal design L9 (3^3^) can be seen in Table 1.

### 2.5. Test of Shear Strength of Plywood

The plywood was conditioned in the laboratory for 1 day. It was then cut into specimens according to the Chinese national standard (GB/T 17657-1999), with dimensions of 100 mm × 25 mm for the test of dry and wet shear strength, which were conducted on a WDS-50KN mechanical testing machine. There was a bonded area of 25 mm × 25 mm for each specimen. The test for dry and wet shear strength made reference of the Chinese national standards GB/T 9846.3-2004 and GB/T 17657-1999, respectively. According to GB/T 17657-1999, as a faster testing method, the specimens were immersed in boiling water for 3 h, the final wet shear strength has to equal 0.9 of the remaining strength. Both the final dry and the wet shear strengths were the mean results of 8–10 tests on independent specimens.

### 2.6. ^13^C-NMR

The 400 μL liquid samples of PG, PF and PFG were mixed well with 200 μL DMSO-d6 for ^13^C-NMR determination. The spectra were obtained on a Bruker AVANCE 600 NMR spectrometer (Bruker, Billerica, MA, USA) using a 12-μs pulse width (90°). The relaxation delay was 6 s. To achieve a sufficient signal-to-noise ratio, inverse-gated proton decoupling method was applied. The spectra were taken at 150 MHz with 400–600 scans accumulated.

### 2.7. Differential Scanning Calorimetry (DSC)

DSC was conducted on soy-based adhesives with or without 9% cross-linkers (PF and PFG). The test was performed on a Perkin-Elmer DSC calorimeter with type DSC 204F1 purchased from Rodgau, Germany. The specimens were heated from 30 to 230 °C under a heat rate of 15 K/min, 20 K/min, 25 K/min, and 30 K/min, respectively, and the thermal changes were recorded. The software used for data analysis was PYRISTM Version 4.0.

## 3. Results and Discussion

### 3.1. The Performance of PFG

The performance of PFG is shown in Table 2. As seen from Table 2, PFG had a lower content of free formaldehyde and free phenol than PF resin, which indicated that when compared with PF resin, PFG showed lower toxicity and was more suitable to be used as the cross-linker for soy-based adhesive.

### 3.2. The Performance of Soy-Based Adhesive Cross-Linked by PF and PFG

Table 3 presents the dry and wet shear strength results of plywood specimens with soy based-adhesive cross-linked by PF or PFG. During the tests on shear strength, only those results with a rupture of wood higher than 75% were thought to be valid and were recorded. As seen from Table 3, the soy-based adhesive without any cross-linker showed no water resistance, as recorded in other reports. Both PF and PFG could be used as a cross-linker for soy-based adhesive to improve its water resistance, indicated by the non-zero wet shear strength of all of the soy-based plywood specimens with cross-linkers. Also seen from Table 3, less PFG than PF was needed to guarantee the wet shear strength of soy-based plywood to satisfy the requirement of the Chinese national standard for type I plywood (GB/T 9846.3-2004, ≥0.70 MPa). The wet strength of soy-based plywood specimens with 9% and 11% PF was lower than that with the same addition amounts of PFG. With 13% PFG, the maximum wet shear strength in boiling water of the soy-based plywood was 1.14 MPa. The viscosity of the final mixture of the soy-based adhesive might be a reason for the inconsistent increase of the wet shear strength with more PFG. After all, the viscosity had a great effect on the interface between the wood and adhesive. The viscosity was observed to decrease after the addition of PF or PFG because of the relatively lower viscosity of PF and PFG than that of the soy-based adhesive. Both PF and PFG improved the dry shear strength of soy-based plywood. Similar to the wet shear strength, it seemed that PFG was a more effective cross-linker for the soy-based adhesive than PF to improve the plywood’s dry strength.

### 3.3. The Chemical Structure of PF and PFG

**Figure 1:**
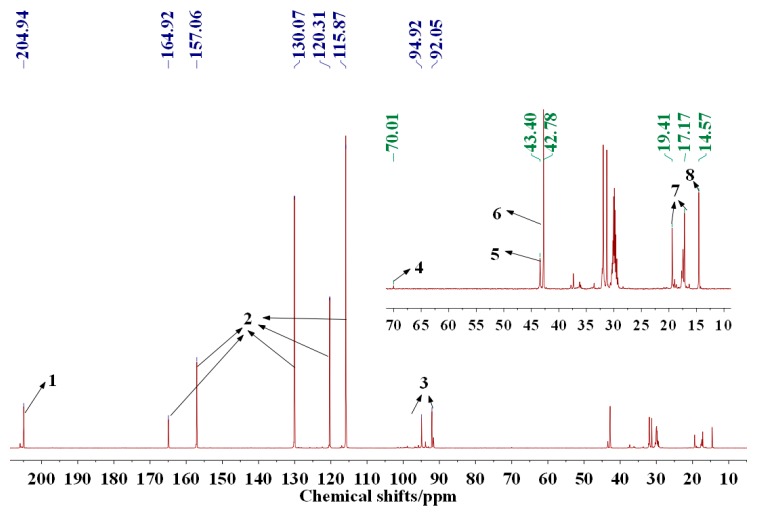
^13^C-NMR spectrum of phenol-glutaraldehyde resin.

To explain why soy-based adhesive cross-linked with PFG showed better shear strength than PF, the chemical structures of PF and PFG were analyzed in this work. Firstly, see the ^13^C-NMR spectrum of phenol-glutaraldehyde (PG) resin (Figure 1). Shown to the top right is the magnified spectrum in the range of 5–75 ppm.

According to the assignment of the labelled peaks outlined in Table 4, the chemical reaction between phenol and glutaraldehyde and the two main possible reaction products could be identified as peak 4 and 6.

**Figure 2:**
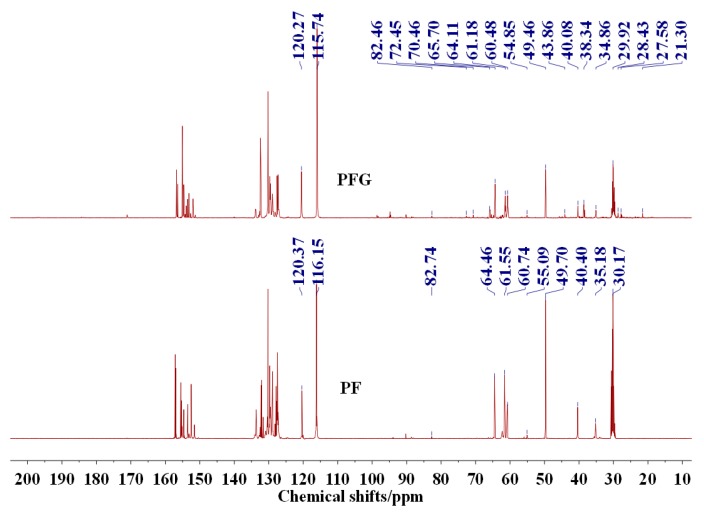
^13^C-NMR spectra of PFG and PF resin.

PFG and PF showed similar ^13^C-NMR results, as seen in Figure 2. The peaks at 60–65 ppm for both PFG and PF samples could be assigned to the hydroxymethyl groups created from the addition reaction between phenol and aldehyde. Compared with PF resin, the ortho hydroxymethyl groups decreased in PFG resin, while the para ones increased. The new peak at 38.5 ppm that appeared in PFG resin could be assigned to the methylene groups from PG [17].

In all, glutaraldehyde reacted with phenol, which resulted in a lower content of free phenol and free formaldehyde in PFG resin than in PF resin. At the same time, the more flexible chemical structure of PFG resin might be responsible for the better mechanical properties and water resistance of the soy-based adhesive cross-linked with it.

### 3.4. Optimization of the Preparation Procedure of Soy-Based Plywood with PFG

The dry and wet shear strength of the soy-based adhesive with 9% PFG obtained from the orthogonal design experiments can be seen in Table 5. The range analysis and the variance analysis of the dry and wet shear strength are also given in Table 6 and Table 7, respectively. As seen from the results, the press temperature had significant effects on both dry and wet strength of the soy-based adhesive. The press time showed significant effects only on the wet strength. The resin loading showed no obvious effects on dry or wet shear strength. Since all the dry shear strengths under the designed press conditions were higher than the relative standard requirement (≥0.70 MPa), the effects of the press procedures on the wet shear strength seemed more important than on the dry strength. Apparently, the press temperature for soy-based adhesive with PFG had to be higher than 140 °C to guarantee both dry and wet strength to be higher than 0.70 MPa. With the increase of the press temperature, press time and resin loading, all of the wet shear strengths increased. When considering both the mechanical properties and the cost of the soy-based plywood, the optimum press temperature was 160 °C, press time 4 min and resin loading 320 g·m^−2^. A verification experiment was done in this study. Under the optimized preparation procedure, that was to say, under 160 °C for 4 min with a resin loading of 320 g·m^−2^, the dry shear strength of the soy-based plywood was 2.11 MPa and the wet shear strength was 0.82 MPa.

### 3.5. DSC Analysis of Soy-Based Adhesive with Cross-Linker PF or PFG

**Figure 3:**
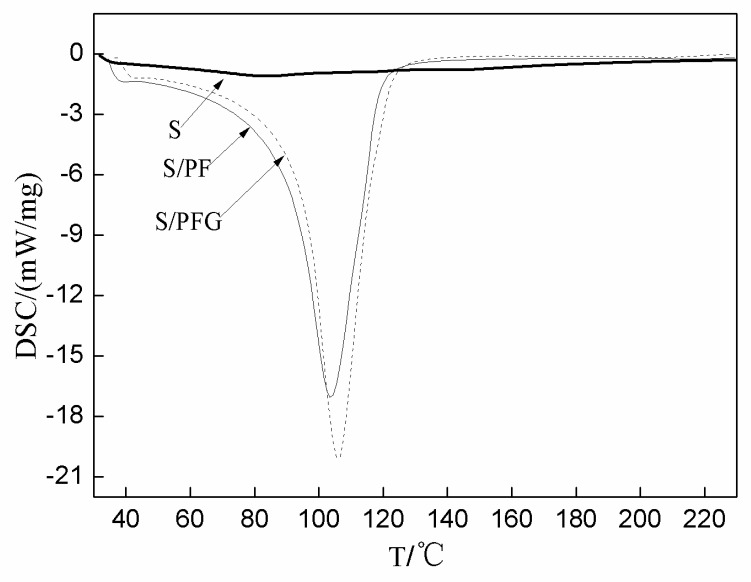
Differential Scanning Calorimetry spectra of soy-based adhesive (S) with or without cross-linker PF or PFG.

Figure 3 shows the DSC spectra of soy-based adhesive without a cross-linker (S) and with cross-linker PF or PFG (S/PF and S/PFG). The heating rate was 15 K/min. As seen from Figure 3, there was no obvious endothermic or exothermic peak for the soy-based adhesive itself, which indicated that the soy protein and other non-protein ingredients in the adhesive would not be cross-linked by themselves. Meanwhile, for both the samples with PF and PFG an exothermic peak at 110–120 °C was shown, which confirmed the reaction between the soy-based adhesive and PF or PFG. As seen from Figure 3, to guarantee the complete curing of the modified soy-based adhesives with PF and PFG, the press temperature had to be higher than its curing temperature, around 110 °C. At the same time, the difference of the peak temperature (Tp) indicated some difference of its curing.

**Figure 4:**
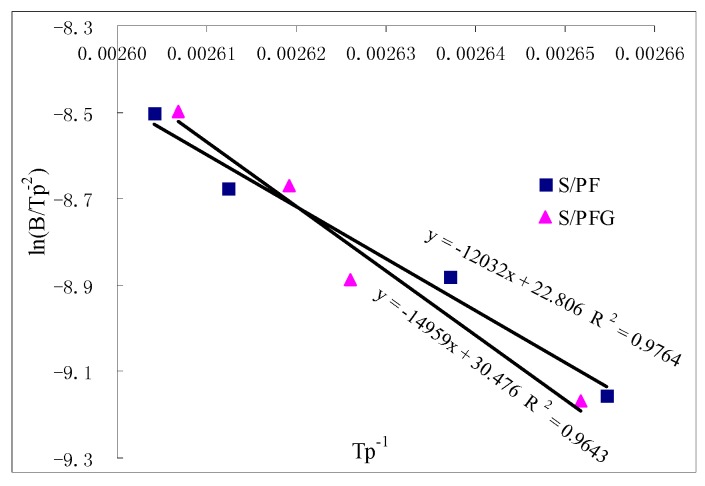
The relationship between 1/Tp and ln(β/Tp^2^) for samples S/PF and S/PFG.

Activation energy (Ea) is a key factor to determine a reaction. In this work, the DSC spectra of samples S/PF and S/PFG were measured under different heat rates (β), specifically, 15 K/min, 20 K/min, 25 K/min and 30 K/min to calculate the Ea according to the Kissinger formula by the slope of the straight line determined by 1/Tp and ln(β/Tp^2^) [18,19]. The Tp obtained from DSC spectra under different heat rates and the calculated Ea for samples S/PF and S/PFG are given in Table 8. The relationship between 1/Tp and ln(β/Tp^2^) for the calculation on Ea of samples S/PF and S/PFG are given in Figure 4. As seen from Table 8, the calculated Ea of sample S/PFG (124.4 KJ/mol) was higher than that of S/PF (100.0 KJ/mol). This implies that a higher press temperature might be required for the soy-based adhesive cross-linked with PFG than with PF to achieve a complete curing. Although the press temperature could not determine the final mechanical performances of the soy-based adhesives, if the press temperature was too low and the adhesive could not cure completely, the mechanical performances of the adhesive would be poor. Therefore, the press temperature would affect the final mechanical performance greatly, which is in accordance with the test results in Table 5.

In all, the test on the performance of the soy-based adhesives cross-linked with PF and PFG proved that PFG could be used as a cross-linker for soy-based adhesives. The preparation procedures for the soy-based plywood was optimized to guide the application of the soy adhesive with PFG. The ^13^C-NMR and DSC tests gave some information on the chemical structure of the cross-linkers and the curing characteristics of the modified adhesive, respectively.

## 4. Conclusions

To prepare a low-formaldehyde soy-based adhesive with good water resistance, phenol-formaldehyde modified with glutaraldehyde (PFG) with lower free phenol and free formaldehyde contents was used to cross-link soy-based adhesive. The results showed that the mechanical properties and water resistance of the soy-based plywood with PFG was better than that of the plywood with the same amount of PF resin. The reaction between phenol and glutaraldehyde was proved by ^13^C-NMR. With the optimized preparation procedures for soy-based plywood, that is to say, press temperature 160 °C, press time 4 min and resin loading 320 g·m^−2^, a type I plywood panel was prepared with 9% PFG as the soy-based adhesive’s cross-linker. The DSC results confirmed the cross-linking reaction between the soy-based adhesive and PFG or PF. The activation energy of the soy-based adhesive with cross-linker PFG was higher than that with PF resin.

## Figures and Tables

**Table 1:** Orthogonal experiment design.

Levels	Factors		
	Press temperature/°C	Press time/min	Resin loading/g·m^−2^
1	140	3	280
2	160	4	320
3	180	5	360

**Table 2:** Performance of phenol-formaldehyde resin modified by glutaraldehyde.

Samples	Viscosity/mPa·s	Solid content/%	Content of free formaldehyde/%	Content of free phenol/%
PF	28.8	44.24	0.28	2.51
PFG	25.5	46.02	0.18	1.56

**Table 3:** The performance of soy-based adhesives with cross-linker phenol-formaldehyde or phenol-formaldehyde-glutaraldehyde resin.

Cross-linker	Dry shear strength/MPa	Wet shear strength in boiling water/MPa
Without a cross-linker	1.68 (0.23)	-
9% PF	2.05 (0.11)	0.63 (0.03)
11% PF	2.02 (0.20)	0.86 (0.06)
9% PFG	2.18 (0.34)	0.82 (0.12)
11% PFG	2.28 (0.34)	1.01 (0.12)
13% PFG	2.19 (0.23)	1.14 (0.08)
15% PFG	2.14 (0.14)	1.01 (0.08)

**Table 4:** The assignment of ^13^C-NMR spectrum of PG resin.

No.	Chemical shift/ppm	Assignment
1	204–206	–CHO
2	116, 120, 130, 157, 165	Carbon from benzene ring
3	90–100	CHO(CH_2_)_3_CH_2_OH
4	70	Ph–CH(OH)CH_2_CH_2_CH_2_CHO or Ph–CH(OH)CH_2_CH_2_CH_2_CH(OH)–Ph
5	43	CHOCH_2_CH_2_CH_2_CHO
6	42	Ph–CH(OH)CH_2_CH_2_CH_2_CHO or Ph–CH(OH)CH_2_CH_2_CH_2_CH(OH)–Ph
7	17–19	Ph–CH(OH)CH_2_CH_2_CH_2_CHO or Ph–CH(OH)CH_2_CH_2_CH_2_CH(OH)–Ph
8	14	CHOCH_2_CH_2_CH_2_CHO

**Table 5:** Dry and wet strength of soy-based plywood with cross-linker PFG and their range analysis.

Trial number	Press temperature/°C	Press time/min	Resin loading/g·m^−2^	Dry shear strength/MPa	Wet shear strength (100 °C)/MPa
1	140	3	280	2.60 (0.15)	0.41 (0.10)
2	140	4	320	2.67 (0.3)	0.51 (0.06)
3	140	5	360	2.59 (0.2)	0.59 (0.05)
4	160	3	320	2.29 (0.1)	0.74 (0.05)
5	160	4	360	2.33 (0.0)	0.82 (0.05)
6	160	5	280	2.05 (0.2)	0.86 (0.06)
7	180	3	360	2.26 (0.3)	0.80 (0.04)
8	180	4	280	1.68 (0.1)	0.87 (0.07)
9	180	5	320	2.07 (0.2)	0.88 (0.08)
Dry shear strength					
K1	2.62	2.38	2.11	—	
K2	2.22	2.23	2.34	—	
K3	2.00	2.24	2.39	—	
R	0.62	0.15	0.28	—	
Wet shear strength in boiling water					
K1	0.50	0.65	0.71	—	
K2	0.81	0.73	0.71	—	
K3	0.85	0.78	0.74	—	
R	0.35	0.13	0.03	—	

**Table 6:** The variance analysis of the orthogonal experiments on dry shear strength.

Factors	Sum of squares of deviations (DEVSQ)	Degree of freedom (DOF)	Mean square error (MSER)	Significance
Press temperature	0.586	2	14.293	*
Press time	0.046	2	1.122	
Resin loading	0.137	2	3.341	
Error	0.04	2		

F0.05 (2,2) = 19; * means significance in 0.05 level.

**Table 7:** The variance analysis of the orthogonal experiments on wet shear strength.

Factors	DEVSQ	DOF	MSER	Significance
Press temperature	0.214	2	214.000	**
Press time	0.025	2	25.000	*
Resin loading	0.001	2	1.000	
Error	0.000	2		

F0.05 (2,2) = 19; **means the factor is very significant in 0.05 level; * means the factor is significant in 0.05 level.

**Table 8:** The Tp obtained from the DSC spectra and the calculated Ea for samples S/PF and S/PFG.

Sample	Tp (K)				Ea/KJ/mol
	15 K/min	20 K/min	25 K/min	30 K/min	
S/PF	376.7	379.2	382.8	384	100.0
S/PFG	379	380.8	381.8	383.6	124.4
